# Ocular toxicity associated with antibody-drug conjugates in cancer therapy: a comprehensive review

**DOI:** 10.3389/fimmu.2026.1698458

**Published:** 2026-04-27

**Authors:** Junxiang Zhou, Haoyue Wang, Hua Xie, Xinyi Xu, Hongtao Xiao

**Affiliations:** 1 Department of Pharmacy, Sichuan Clinical Research Center for Cancer, Sichuan Cancer Hospital & Institute, Sichuan Cancer Center, University of Electronic Science and Technology of China, Chengdu, China; 2 Department of Medical Oncology, Sichuan Clinical Research Center for Cancer, Sichuan Cancer Hospital & Institute, Sichuan Cancer Center, University of Electronic Science and Technology of China, Chengdu, China; 3 Department of Pharmacy, Public Health Clinical Center of Chengdu, Chengdu, China

**Keywords:** adverse event, antibody-drug conjugate, cancer, clinical trial, ocular toxicity, pharmacovigilance

## Abstract

Ocular toxicity from anti-cancer drugs is frequently overlooked, largely because it is non-life-threatening, whereas clinical priorities often center on adverse events that pose immediate risks to survival. Antibody-drug conjugates (ADCs) have emerged as an important therapeutic modality in oncology, offering potent cytotoxicity against tumor cells through the targeted delivery of payloads. However, this potency also introduces a spectrum of toxicities, among which ocular toxicity has gained increasing attention. ADC-induced ocular toxicity can impair visual function and significantly compromise both quality of life and treatment adherence. This review compiles data on ocular toxicity reported during clinical development and postmarketing surveillance of all currently approved ADCs. This review analyzes the potential mechanisms underlying ADC-associated ocular toxicity and provides a detailed summary of the reported ocular adverse events and their incidence across approved ADCs. It also highlights the significance of effective toxicity management for early detection, monitoring, and treatment of ocular toxicity. Finally, this review explores potential strategies to mitigate ADC-associated ocular toxicity from a drug development perspective. A more comprehensive understanding of ocular toxicity, combined with innovative approaches to target selection, linker design, payload optimization, as well as the biological processes underlying *in vivo* interactions between ADCs and tissues, may enable the reduction of ocular toxicity and improvement of clinical outcomes in future ADC therapy.

## 1 Introduction

Over the past two decades, the approval of numerous novel anti-cancer therapies, including targeted agents and immunotherapy agents, has significantly advanced cancer treatment and improved patient survival outcomes. Targeted therapies selectively act on tumor cells by interfering with specific molecular pathways or protein targets, allowing for precise cytotoxic effects while minimizing damage to normal tissues. This approach reduces off-target toxicity and helps to avoid many of the adverse effects and poor tolerability associated with traditional cytotoxic chemotherapy. However, these therapies remain associated with a range of adverse events (AEs) in clinical practice. Notably, several anti-cancer agents have been implicated in ocular toxicities. Agents known to induce such toxicities include epidermal growth factor receptor (EGFR) inhibitors, breakpoint cluster region-abelson (BCR-ABL) inhibitors, immune checkpoint inhibitors (ICIs), anaplastic lymphoma kinase (ALK) inhibitors, antibody-drug conjugates (ADCs), among others (1, 2). The eye is a visual system structure composed of the eyeball and its associated adnexal organs. Although the eye is protected by the blood-ocular barrier, several anti-cancer agents have been shown to induce ocular toxicity. This may manifest as either functional or structural ocular damage, ranging from transient visual disturbances to permanent vision loss, significantly impacting daily functioning and quality of life (3). With the widespread use of ADCs, the safety risks posed by ADCs have become increasingly prominent. Ocular toxicity has emerged as an important dose-limiting toxicities for several ADCs due to its relatively high incidence and the potential risk of significant visual impairment (4). In clinical trials of several ADCs, ocular AEs have led to premature treatment discontinuation in more than 50% of patients (5, 6), highlighting the clinical importance of this complication.

The concept of ADC, often referred to as “magic bullets”, was first proposed in 1908 by Paul Ehrlich, a German physician and scientist (7). ADC is a macromolecular monoclonal antibody-based drug composed of three key components:(i) an antibody that selectively targets antigens expressed on the surface of cancer cells, ensuring precise delivery of the therapeutic agent; (ii) a cytotoxic drug/payload that induces cell death by damaging DNA or inhibiting cell division; and (iii) a linker that covalently connects the antibody to the payload, enabling stable and controlled drug release. The mechanism of ADC primarily involves the binding of the antibody component to target antigens specifically expressed on the surface of cancer cells. Following antigen recognition, the ADC is internalized into the cell via endocytosis, forming early endosomes that subsequently mature into late endosomes and fuse with lysosomes. Within the lysosomal environment, the payload is released through chemical or enzymatic cleavage of the linker, ultimately inducing apoptosis or cell death by targeting DNA or disrupting microtubule function (8). When the released payload is membrane-permeable or capable of crossing cell membranes, it may enhance the therapeutic efficacy of the ADC through a bystander effect. Additionally, this effect may alter the tumor microenvironment, thereby potentially further augmenting the cytotoxic activity of the ADC (8, 9).

ADC exploits the targeting precision and high specificity of monoclonal antibodies to deliver payload directly to tumor sites, enabling the therapeutic use of highly potent agents that would otherwise be undruggable for systemic administration due to their toxicity. Any component of ADC plays a crucial role in determining efficacy and safety of the drug. Among these, the payload is the primary driver of both therapeutic potency and toxicity. The cytotoxic payload has been recognized as a major contributor to ADC-associated ocular toxicity. At present, ADC payloads are mainly divided into two categories: microtubule inhibitors and DNA-damaging agents (7). Microtubule inhibitors, such as auristatin and maytansinoid, disrupt microtubule dynamics, inducing mitotic arrest and thereby halting the rapid proliferation of tumor cells (10, 11). DNA-damaging agents act independently of the cell cycle by binding to DNA and inducing strand breaks or other forms of damage, ultimately triggering cell death. These include DNA-fragmentation agents, DNA-alkylating compounds, and topoisomerase I/II inhibitors. Common used payloads in this category include pyrrolobenzodiazepine, duocarmycin, calicheamicin, SN-38, and DXd (12, 13). As ADCs become more widely used in clinical practice, it is increasingly important to remain vigilant about their toxicity profiles, especially ocular toxicity. To date, the U.S. Food and Drug Administration (FDA) has required boxed warnings in the prescribing information for belantamab mafodotin, tisotumab vedotin, and mirvetuximab soravtansine due to their notable ocular AEs. However, current understanding of ADC-induced ocular toxicity remains limited, and there is a lack of systematic knowledge regarding its underlying mechanism, clinical manifestations, and toxicity management.

Visual function is closely related to the quality of life in patients. Impaired vision can severely restrict the ability to perform daily activities and work, resulting in increased dependence and psychological distress. For cancer patients, even mild ocular AEs can significantly reduce treatment adherence. Ocular toxicity, as a generally non-fatal AE, is often not given sufficient attention in conventional cancer therapy. Ocular toxicity is a characteristic AE of ADCs, it is particularly critical to fully recognize, understand and manage the ocular toxicity associated with ADCs. This article comprehensively reviews the mechanism of ocular toxicity caused by ADCs, clinical manifestations of ocular toxicity observed in both clinical trials and postmarketing real-world settings for approved ADCs, and management strategies for ocular toxicity. It highlights the need for identifying ocular AEs and also offers insights to guide the development of next-generation ADCs.

## 2 Methods

A literature search was conducted using PubMed and Cochrane Central Register of Controlled Trials. Search terms included the generic names of ADCs, development code names used during clinical trials, and trial identifiers. The search was restricted to clinical trial to identify ocular toxicities reported during investigational phases. Additional searches were performed using both generic and brand names of ADCs to capture postmarketing ocular AEs reported in studies based on the FDA Adverse Event Reporting System (FAERS), case reports, case series, and real-world studies. In this review, all ADCs approved globally as of May 2025 were identified, and the literature search was limited to studies published in English up to May 2025. Studies reporting any ocular AEs associated with ADCs were included, whereas those evaluating ADCs in combination with ICIs, EGFR inhibitors, human epidermal growth factor receptor 2 (HER2) inhibitors, ALK inhibitors, BCR-ABL inhibitors, phosphoinositide 3-kinase/protein kinase B inhibitors and poly (ADP-ribose) polymerase inhibitors were excluded. No restrictions were applied regarding tumor type, study population, prior treatment regimens, or other factors. A detailed search strategy is provided in **Supplementary Table 1**. Relevant information was also retrieved from official regulatory sources, including ClinicalTrials.gov and the FDA website. Google Scholar was searched as a supplementary source to identify gray literature.

## 3 Mechanisms of ocular toxicity associated with ADCs

Ocular toxicity associated with ADCs involves multiple anatomical structures of the eye and presents with diverse clinical manifestations. Although the underlying mechanisms remain incompletely understood, the most widely accepted hypothesis involves the formation of bilateral microcystic-like epithelial changes originating in the peripheral cornea and migrating centrally, ultimately leading to symptoms such as dry eye and blurred vision (14). This toxicity is thought to arise primarily from off-target effects. One contributing factor is the *in vivo* instability of some ADCs and the premature release of their cytotoxic payloads. If the linker–payload complex lacks sufficient stability, free drug may be released into the circulation too early, increasing exposure to non-target tissues such as the eye (15). The physicochemical characteristics of ADCs, especially the charge and hydrophobicity, can influence tissue distribution and diffusion of payload. For example, ADCs with a positive charge may be more readily internalized by periorbital cells through charge-mediated uptake, which can contribute to ocular toxicity (16, 17). Another proposed mechanism is the bystander effect, whereby payloads such as monomethyl auristatin E (MMAE) or SN-38 diffuse from targeted cells into adjacent tissues or re-enter systemic circulation. Due to the dense vasculature of ocular tissues, such diffusion may lead to the onset of ocular toxicity (18, 19). In addition, on-target, off-tumor toxicity can occur when ADC target antigens are expressed at low levels in normal ocular tissues. For instance, the ocular adverse effects observed with Tisotumab Vedotin are thought to result from binding to tissue factor (TF), which is expressed in the conjunctiva (20). Other commonly targeted antigens, including HER2, trophoblast cell surface antigen 2 (Trop-2), Nectin-4, folate receptor alpha (FRα), CD19, CD22, CD33, and CD79b, have also been detected in ocular tissues, making them susceptible to antigen-mediated uptake and potential damage (21). Nonspecific uptake mechanisms may also play a role. ADCs can be internalized by ocular cells through macropinocytosis, leading to drug accumulation and toxicity (15). Notably, one study has demonstrated that modulating this pathway may reduce ocular exposure and help mitigate toxicity (16).

Based on the drug-release mechanisms, ADC linkers can be broadly divided into cleavable and non-cleavable types. The ADCs designed based on cleavable linker primarily rely on the release of payloads triggered by tumor-associated acidic microenvironments, lysosomal protease activity, or thiol-disulfide exchange reactions (22–24). In contrast, ADCs incorporating non-cleavable linker must undergo lysosomal internalization, where enzymatic degradation of the antibody component generates active payloads conjugated to charged lysine or cysteine residues. Compared with this mechanism, cleavable linker-based ADCs are more vulnerable to environmental factors, which may lead to premature payload release and subsequent off-target toxicity. The nature of the payload also influences toxicity profiles. Ocular toxicity has been most commonly associated with ADCs that incorporate monomethyl auristatin F (MMAF) or DM4 as payload. Available evidence indicates that ocular adverse effects linked to belantamab mafodotin and tisotumab vedotin are primarily driven by off-target injury to corneal epithelial cells caused by auristatin-type cytotoxins, including MMAF and MMAE. In belantamab mafodotin, MMAF disrupts microtubule assembly and directly damages corneal epithelial cells. Moreover, its charged metabolites exhibit limited membrane permeability, leading to intracellular accumulation and further enhancing ocular toxicity. This results in off-target apoptosis of corneal epithelial cells, which manifests clinically as microcyst-like epithelial changes or keratopathy observable by slit-lamp examination (25, 26). The ocular toxicity associated with tisotumab vedotin may be attributed to the expression of TF in the conjunctiva, which facilitates the targeted delivery of the payload MMAE to conjunctival tissues, resulting in epithelial cell death and local inflammation (27, 28). In addition, the cleavable linker of tisotumab vedotin enables a bystander effect, further amplifying inflammatory responses in adjacent conjunctival cells (29, 30). The ocular AEs observed with mirvetuximab soravtansine was related to the off-target effect of the payload DM4. This agent exerts antimitotic activity on dividing cells within the corneal epithelium, disrupting epithelial integrity and leading to microcyst-like epithelial changes (20, 25). Ocular toxicity of trastuzumab deruxtecan may be caused by the topoisomerase I inhibitor SN-38 via a bystander effect involving systemic or local diffusion into ocular tissues. Given the rapid turnover and high metabolic activity of the conjunctival and corneal epithelium, these tissues may exhibit heightened sensitivity to topoisomerase I inhibition, contributing to the ocular AEs (19, 31).

## 4 Ocular toxicity associated with approved ADCs

**Figure 1:**
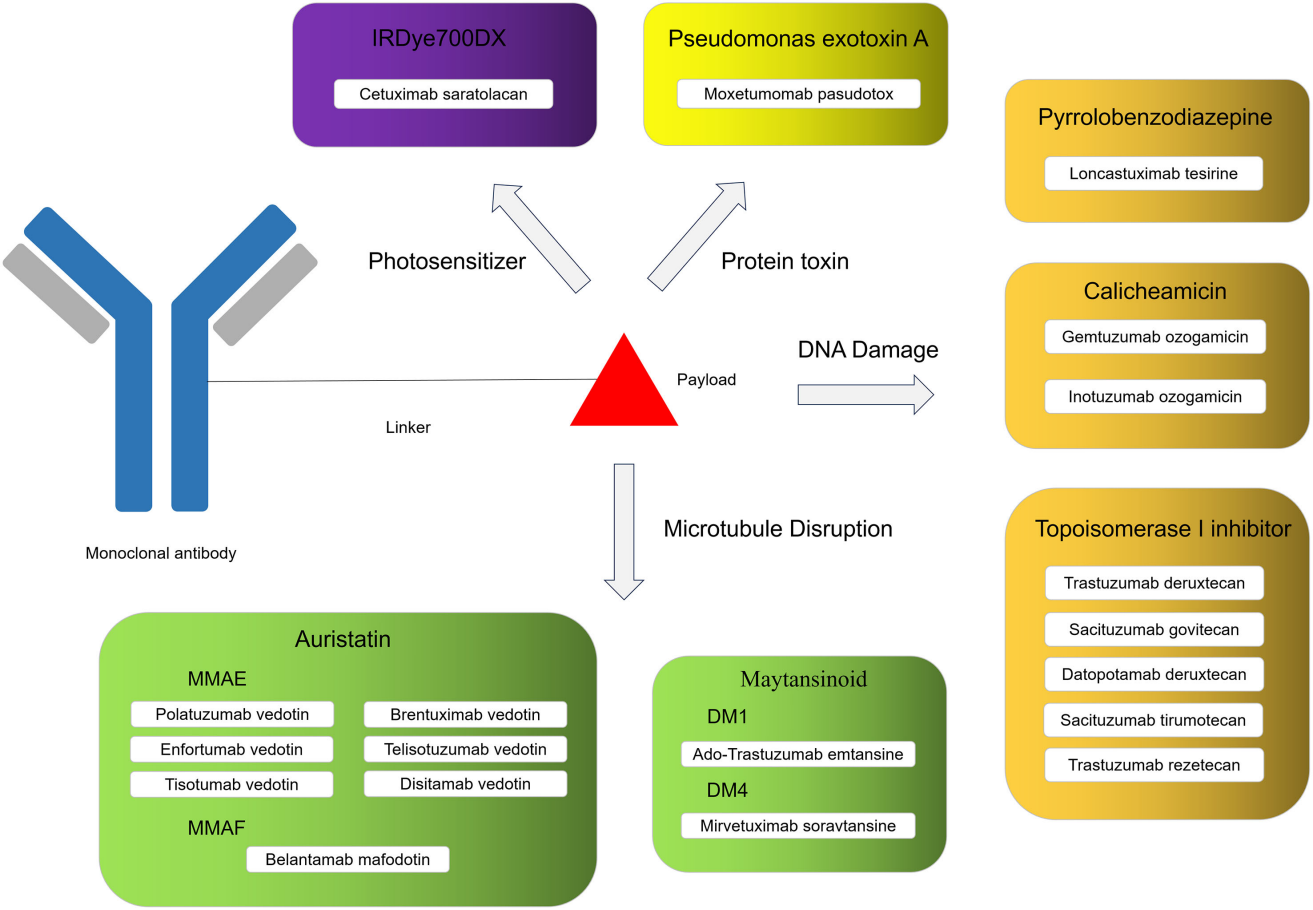
Approved ADCs classification by payload.

**Figure 2:**
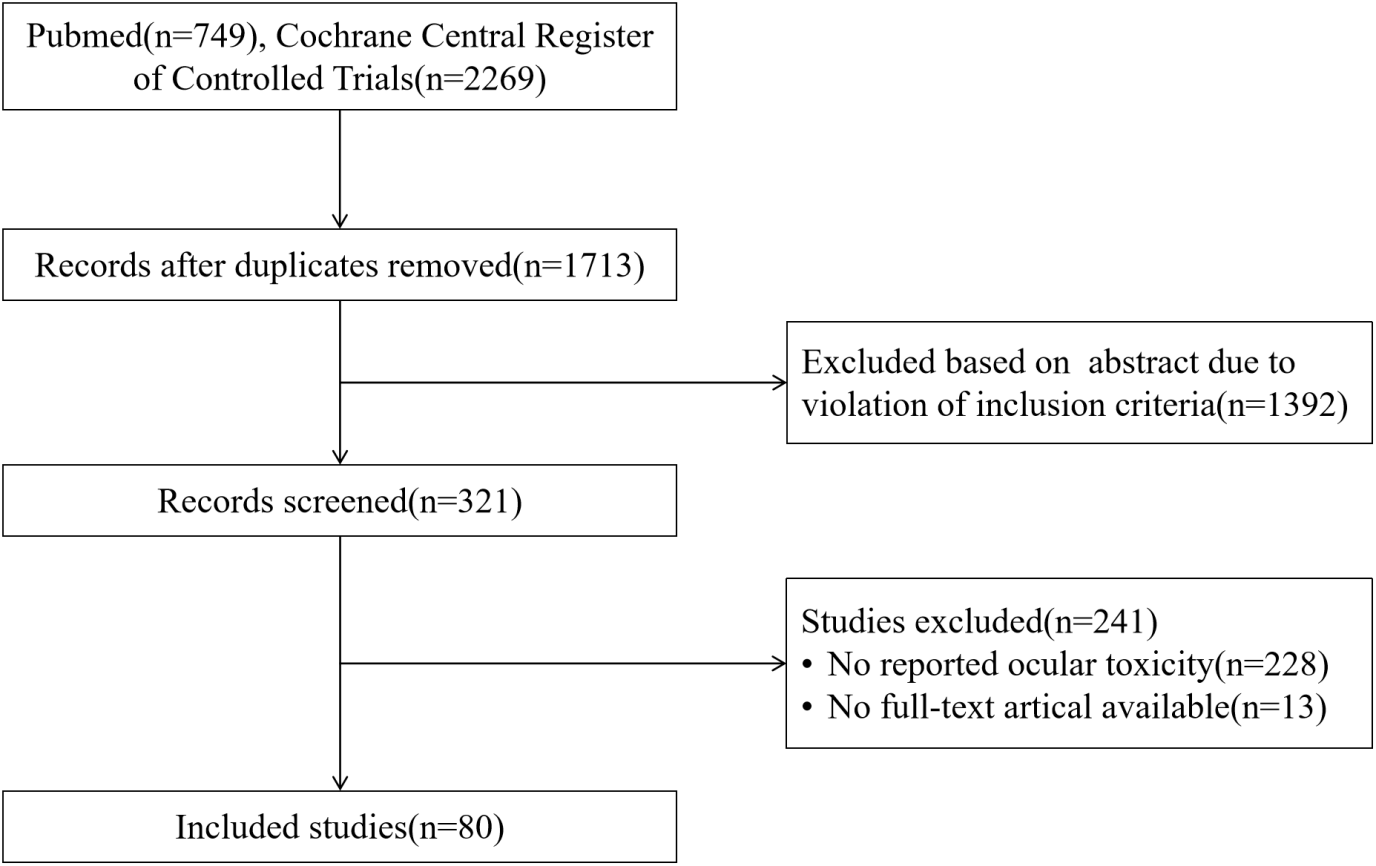
Flowchart of literature selection.

As of now, a total of 19 ADCs have been approved globally for the treatment of cancer (**Figure 1**), These agents are indicated for a range of malignancies, such as leukemia, lymphoma, head and neck cancer, breast cancer, lung cancer and ovarian cancer. After literature screening (**Figure 2**), a total of 80 eligible studies were included, comprising 43 clinical trials and 37 postmarketing studies. Ocular AEs associated with ADCs reported in the literature are summarized in **Table 1**. Detailed information on ocular AEs observed with ADCs in clinical trials and postmarketing studies is provided in **Supplementary Tables 2**, **3**. The lower bound of the 95% confidence interval for information component (IC025) values are provided for safety signals identified from the FAERS data mining; incidence rates are reported for AEs observed in real-world studies; and specific events are listed for those documented in case reports. Ocular toxicities of ADCs are described below according to the type of payload.

**Table 1:** Ocular AEs associated with ADCs.

ADC	Standard intravenous dosage^δ^	Ocular AEs
Ado-Trastuzumab emtansine	3.6 mg/kg, every 3 weeks	Dry eye, cataract, punctuate keratitis, increased lacrimation, blurred vision/visual impairment, conjunctivitis, excessive eye blinking^*^, blindness^*^, retinal detachment^*^, asthenopia^*^, ulcerative keratitis^*^, abnormal sensation in eye^*^, scintillating scotoma^*^, hypermetropia^*^, corneal deposits^*^, eye haemorrhage^*^, diplopia
Mirvetuximab soravtansine	6 mg/kg, every 3 weeks	Visual impairment, keratopathy, dry eye, cataract, photophobia, blurred vision, visual acuity reduced, eye pain, keratitis, vitreous floaters
Polatuzumab vedotin	1.8 mg/kg, every 3 weeks for 6 cycles	Visual acuity reduced, dry eye, blurred vision, age-related macular degeneration^*^, choroidal neovascularisation^*^
Enfortumab vedotin	1.25 mg/kg (up to a maximum of 125 mg), days1,8,15, every 4 weeks	Dry eye, blurred vision, corneal disorders, increased lacrimation, keratitis^*^, eye discharge^*^, eye haemorrhage^*^, abnormal sensation in eye^*^, blepharitis^*^, xeropthalmia^*^, giant papillary conjunctivitis^*^, conjunctivitis, abducens paresis
Tisotumab vedotin	2 mg/kg (up to a maximum of 200 mg), every 3 weeks	Conjunctivitis, dry eye, ulcerative keratitis, blepharitis, keratitis, conjunctival ulcer, blurred vision, vital dye staining cornea present, conjunctival hyperemia, conjunctival scar, corneal irritation, corneal thinning, erythema of eyelid, eye irritation, eye nevus, meibomianitis, punctate keratitis, trichiasis, visual acuity reduced, conjunctivitis allergic, scleritis, hordeolum, eye discharge, eye pruritis, eye pain, increased lacrimation, cataract, ocular hyperaemia^*^, ectropion^*^, symblepharon^*^, corneal deposits^*^, corneal opacity^*^, conjunctival subepithelial fibrosis, pseudomembranous conjunctivitis, infectious keratitis
Brentuximab vedotin	1.8 mg/kg (up to a maximum of 180 mg), every 3 weeks	Blindness^*^, papilloedema^*^, retinal haemorrhage^*^, visual acuity reduced transiently^*^, uveitis, panuveitis, Vogt-Koyanagi-Harada-like granulomatous pan uveitis, purtscher-like retinopathy, retinal detachment^#^
Telisotuzumab vedotin	1.9 mg/kg, every 2 weeks	Blurred vision, keratitis, dry eye
Disitamab vedotin	Gastric cancer: 2.5 mg/kg, every 2 weeks Urothelial carcinoma: 2 mg/kg, every 2 weeks	Increased lacrimation, blurred vision, periorbital swelling
Belantamab mafodotin	2.5 mg/kg, every 3 weeks	Blurred vision, dry eye, photophobia, visual impaiment, corneal deposits, corneal irritation, corneal oedema, corneal opacity, diplopia, eye pain, eye pruritus, foreign body sensatlon in eyes, keratitis, keratopathy, limbal stem cell deficiency, photopsia, punctate keratitis, increased lacrimation, night blindness, corneal epithelium changes, exophthalmos, glaucoma, retinal vein occlusion, visual acuity reduced, conjunctival hemorrhage, xerophthalmia, vision loss, epithelial crystal-like deposits, microcystic keratopathy
Loncastuximab tesirine	0.15 mg/kg, every 3 weeks for 2 cycles, 0.075 mg/kg every 3 weeks for subsequent cycles	Cataract
Gemtuzumab ozogamicin	Newly-diagnosed AML: Induction: 6 mg/m^2^, day1, 3 mg/m^2^, day8; Consolidation: 2 mg/m^2^, day1, every 4 weeks, up to 8 continuation courses. Relapsed or refractory AML: 3 mg/m^2^, days1,4,7	Eye haemorrhage, retinal haemorrhage, conjunctival haemorrhage, scleral haemorrhage, eyelid hematoma, eye swelling, periorbital edema
Inotuzumab ozogamicin	0.8 mg/m^2^, day1, 0.5 mg/m^2^, days8,15, for cycle1(3 weeks), followed by 0.5 mg/m^2^, days1,8,15, every 4 weeks or 0.8 mg/m^2^, day1, 0.5 mg/m^2^, days8,15, every 4 weeks, depending on the response to treatment	Conjunctival hemorrhage, eyelid bleeding, eyelid oedema^*^
Trastuzumab deruxtecan	5.4 mg/kg, every 3 weeks or 6.4 mg/kg, every 3 weeks	Dry eye, periorbital edema, eyelid edema, blurred vision, eye hemorrhage, excessive eye blinking^*^, keratitis^*^, punctate keratitis^*^, pinguecula*, blindness^*^
Datopotamab deruxtecan	6 mg/kg, every 3 weeks	Dry eye, increased lacrimation, blepharitis, keratitis, cataract, ulcerative keratitis, conjunctivitis, corneal erosion, corneal lesion, foreign body sensation in eyes, keratopathy, limbal stem cell deficiency, meibomian gland dysfunction, photophobia, punctate keratitis, superior limbic keratoconjunctivitis, blurred vision, visual impairment, xerophthalmia
Sacituzumab govitecan	10 mg/kg, days1,8, every 3 weeks	Periorbital edema, eyelid ptosis^*^, cataract^*^, dry eye^*^, ulcerative keratitis^*^, increased lacrimation^*^, visual impairment^*^, papilledema of optic nerve^*^, photophobia^*^, eye congestion^*^
Sacituzumab tirumotecan	5 mg/kg, every 2 weeks	Dry eye, blurred vision, ophthalmodynia, xerophthalmia
Trastuzumab rezetecan	4.8 mg/kg or 408mg (85 kg or above), every 3 weeks	Ulcerative keratitis, blurred vision, conjunctival hyperemia, increased lacrimation
Moxetumomab pasudotox	0.04 mg/kg, days1,3,5, every 4 weeks	Blurred vision, dry eye, cataract, ocular discomfort/pain, ocular swelling/periorbital edema, conjunctivitis, conjunctival hemorrhage, ocular discharge
Cetuximab saratolacan	640mg/m^2^	Eye disorders, periorbital edema

ADC, antibody-drug conjugate; AEs, adverse events; δ, listed doses are monotherapy recommendations, except for polatuzumab vedotin; AML, acute myeloid leukemia; *, only from FDA Adverse Event Reporting System signal analysis; #, only from Japanese Adverse Drug Event Report database signal analysis.

### 4.1 Maytansinoid

#### 4.1.1 Ado-Trastuzumab emtansine

Ado-Trastuzumab emtansine (T-DM1), a humanized anti-HER2 immunoglobulin G1 (IgG1) antibody, is linked to the microtubule inhibitor DM1 through a stable noncleaved succinimidyl trans-4-(maleimidylmethyl)cyclohexane-1-carboxylate linker. In the phase I trial (32), dose-escalation ranged from 1.2 mg/kg weekly (qw) to 2.9 mg/kg qw. Among 28 HER2-positive metastatic breast cancer(MBC) patients previously treated with trastuzumab, approximately 46.4% reported ocular AEs, with dry eye occurring in 10.7%. Two patients experienced grade 3 AEs, including cataracts, ocular surface disease, and punctate keratitis. In the phase II trial (33), T-DM1 was administered at 3.6 mg/kg every three weeks (q3w) in 112 patients with HER2-positive MBC, with 31.5% of patients experiencing ocular AEs. These primarily manifested as dry eye, increased lacrimation, blurred vision/visual impairment, and conjunctivitis, most of which were grade 1-2, and no severe ocular AEs were reported. The EMILIA trial (34, 35) 36was a phase III clinical study involving 490 patients with HER2-positive, unresectable locally advanced or MBC who received T-DM1 at a dose of 3.6 mg/kg q3w. The blurred vision occurred in 4.5% of patients, conjunctivitis in 3.9%, dry eye in 3.9%, and increased lacrimation in 3.3%. Most ocular toxicities were mild to moderate in severity, with no reported grade 4 ocular AEs. Treatment interruptions or dose modifications due to ocular toxicity were infrequent, with 22.9% of patients requiring dose adjustments (36). In another phase III study, the KATHERINE trial (35, 37), involving 720 patients with HER2-positive early breast cancer treated with T-DM1 3.6 mg/kg q3w, 6% experienced increased lacrimation, 4.5% developed dry eye, 3.9% reported blurred vision, and 3.5% experienced conjunctivitis.

Postmarketing pharmacovigilance studies (38–40) based on the FAERS database have confirmed that T-DM1 is associated with ocular AEs including excessive eye blinking, increased lacrimation, retinal detachment, asthenopia, abnormal sensation in eye, scintillating scotoma, and others. In real-world settings, the median time to onset of ocular toxicity associated with T-DM1 was 43.5 days (41). A case report described a 36-year-old female patient with MBC who developed increased lacrimation after two months of T-DM1 treatment; symptoms improved after two months of four times daily administration of tobramycin 0.3% combined with dexamethasone 0.1% eye drops (42). Another case involved a 67-year-old female breast cancer patient who experienced transient grade 2 blurred vision following the first infusion of T-DM1 (3.6 mg/kg q3w) as second-line therapy. After the second infusion, her blurred vision worsened to grade 3, accompanied by grade 2 diplopia causing dizziness. Symptoms of diplopia resolved four months after cessation of T-DM1, and vision recovered after seven months. The authors attributed this to possible abducens cranial nerve injury (43).

#### 4.1.2 Mirvetuximab soravtansine

Mirvetuximab soravtansine is an ADC targeting FRα, composed of a humanized monoclonal antibody linked to the tubulin-disrupting maytansinoid DM4 via a cleavable disulfide linker. The SORAYA trial (44, 45), a single-arm, open-label phase II study, evaluated mirvetuximab soravtansine at 6 mg/kg q3w in 106 patients with platinum-resistant ovarian cancer. The most frequently reported ocular toxicities were blurred vision and keratopathy, with median onset times of 1.3 months and 1.5 months respectively. Long-term follow-up revealed that 43% of patients experienced visual impairment, 36% developed keratopathy, and 25% reported dry eye; grade ≥3 events occurred in 6%, 9%, and 2% of patients respectively (45). The FORWARD I trial (46), a randomized, open-label phase III study, enrolled 248 patients with platinum-resistant ovarian cancer who received mirvetuximab soravtansine 6 mg/kg q3w. Blurred vision (all grades: 42.0%; grade 3: 2.5%) and keratopathy (all grades: 32.5%; grade 3: 1.2%) were the most common ocular toxicities and the leading causes of treatment delay or dose modification. Other severe ocular AEs included dry eye, reported in 1.2% of patients. The MIRASOL trial (47), another randomized, open-label phase III study, assessed mirvetuximab soravtansine 6 mg/kg q3w in 218 patients with platinum-resistant ovarian cancer. Ocular AEs occurred in 56% of patients, including blurred vision, keratopathy, dry eye, photophobia, cataract, visual acuity reduced, among others. Grade 3 ocular toxicities included keratopathy (9.2%), blurred vision (7.8%), dry eye (3.2%), cataract (3.2%), visual acuity reduced (3.2%), and others. The median time to onset of ocular toxicity was 5.4 weeks. No grade ≥4 events of blurred vision, keratopathy, or dry eye were reported, and no cases of corneal ulceration, perforation, or permanent ocular sequelae were observed.

A retrospective study (48) involving 25 patients with FRα-positive, recurrent, platinum-resistant ovarian cancer treated with mirvetuximab soravtansine reported an ocular toxicity incidence of 64%. Among these patients, 20% experienced treatment delays and 32% required dose modifications. Grade 3 ocular toxicities, including visual acuity reduced and blurred vision, occurred in 16% of patients, although none discontinued treatment due to ocular AEs. In real-world settings, the median time to onset of ocular toxicity was 28 days (49). A case report described a 61-year-old woman with stage IIIC fallopian tube cancer who developed blurred vision, dry eye, keratitis, and cataract after two cycles of mirvetuximab soravtansine (50). Another case report documented corneal toxicity following two treatment cycles in a 68-year-old patient with ovarian cancer (51).

### 4.2 Auristatin

#### 4.2.1 Polatuzumab vedotin

Polatuzumab vedotin is an ADC composed of a CD79b-targeting monoclonal antibody linked to MMAE via a cleavable valine–citrulline dipeptide linker. The GO29365 study (52), a phaseIb/IItrial, evaluated the safety and efficacy of polatuzumab vedotin at 1.8 mg/kg q3w in combination with bendamustine and rituximab in patients with relapsed or refractory diffuse large B-cell lymphoma. In the polatuzumab vedotin expansion cohort, three cases (1.7%) of grade 1 visual acuity reduced were reported, with two cases (1.2%) considered treatment-related. According to the FDA medical review, one case of keratitis was observed among patients receiving polatuzumab vedotin at 1.8 mg/kg, with no reports of ocular deposition. Other nonspecific ocular AEs included dry eye (five cases) and blurred or impaired vision (16 cases) (53). In a real-world study (38), ocular AEs associated with polatuzumab vedotin has been infrequently reported, with age-related macular degeneration and choroidal neovascularization being the most commonly noted manifestations.

#### 4.2.2 Enfortumab vedotin

Enfortumab vedotin is an ADC composed of an antibody targeting Nectin-4 and MMAE, which are linked together by a cleavable valine-glycine dipeptide linker. The EV-103 trial (54), a phase Ib/II clinical study, evaluated the safety and efficacy of enfortumab vedotin (1.25 mg/kg on days 1 and 8 of a 21-day cycle) either as monotherapy or in combination with other agents in patients with locally advanced or metastatic urothelial cancer. In cohort K, among 76 patients treated with enfortumab vedotin in combination with pembrolizumab, ocular AEs occurred in 26.3%, with rates of dry eye and blurred vision reported at 25% and 2.6%, respectively. Among 73 patients receiving enfortumab vedotin monotherapy, the incidence of ocular AEs was 28.8%, including dry eye in 28.8%, blurred vision in 6.8%, and corneal disorders in 5.5%. In EV-201 trial (55), among 125 patients with locally advanced or metastatic urothelial cancer treated with enfortumab vedotin at 1.25 mg/kg on days 1, 8, and 15 of a 28-day cycle, 23% experienced dry eye, 14% reported increased lacrimation, and 15% developed blurred vision. In EV-301 trial (56), ocular AEs occurred in 18.6% of 296 patients, predominantly mild to moderate in severity. The incidence of dry eye was 15.9%, with a median onset of 1.9 months; blurred vision occurred in 4.1%, with a median onset of 2.4 months; and corneal disorders were reported in 0.7% of patients, with a median onset of 4.3 months. In EV-302 trial (57), 45 patients with locally advanced or metastatic urothelial cancer received enfortumab vedotin (1.25 mg/kg on days 1 and 8 of a 21-day cycle) combined with pembrolizumab (200 mg q3w). The incidence of ocular toxicities of any grade was 35.6% for dry eye, 11.1% for blurred vision, and 2.2% for corneal disorders, all of which were grade 1 or 2. Overall, multiple clinical trials have demonstrated a relatively high incidence of ocular toxicity with enfortumab vedotin, occurring in 44% of patients. The most common symptoms were dry eye in 19% and blurred vision in 14%, with a median time to onset of changes in vision at 94 days (58).

Two pharmacovigilance studies based on the FAERS database (38, 59) identified ocular AEs associated with enfortumab vedotin, including dry eye, eye discharge, corneal disorders, keratitis, abnormal sensation in eye, and others. In real-world studies (60–63), the incidence of eye disorders associated with enfortumab vedotin ranged from 5.6% to 15%, with reported AEs including blurred vision, dry eye, conjunctivitis, and abducens paresis. The median time to onset of ocular toxicities in these real-world settings was 23.5 days (49).

#### 4.2.3 Tisotumab vedotin

Tisotumab vedotin consists of a TF-targeting antibody, MMAE and a cleavable valine-citrulline dipeptide linker. In InnovaTV 201 trial (64, 65), dose-escalation in the phase I ranged from 0.3 to 2.2 mg/kg q3w, and the recommended phase II dose was established as 2 mg/kg q3w. Among patients with recurrent or refractory cervical cancer who did not receive ocular prophylactic measures, the incidence of ocular toxicity reached 80%, with the most common AEs being conjunctivitis, dry eye, ulcerative keratitis, blepharitis, and keratitis. Most ocular AEs were mild to moderate, though a minority of patients experienced severe conjunctivitis. Following the implementation of ocular prophylaxis, the incidence of ocular toxicity declined to 65%, and the rate of conjunctivitis fell from 80% to 28%. In InnovaTV 204 trial (66), 138 ocular AEs were reported in 101 patients with recurrent or refractory cervical cancer treated with tisotumab vedotin 2 mg/kg q3w, corresponding to an overall incidence of 53%. Most AEs were grade 1 or 2 and limited to the ocular surface. The median time to onset was 1.4 months, and the median time remission was 0.7 months. Conjunctivitis was reported in 26% of patients, dry eye in 23%, and keratitis in 11%. Two patients (2%) experienced grade 3 ulcerative keratitis, both of whom discontinued treatment. Dose reductions due to ocular toxicity occurred in 22% of patients. In the dose-expansion phase of InnovaTV 206 study (67), 17 patients with recurrent or metastatic cervical cancer were treated with tisotumab vedotin at 2 mg/kg q3w. Ocular AEs were reported in 35.3% of patients. Conjunctivitis occurred in 17.6%, conjunctivitis allergic in 5.9%, scleritis in 5.9%, hordeolum in 5.9%, and blurred vision in 5.9%. All ocular AEs were grade 1 or 2, with no grade 3 or higher AEs reported. InnovaTV 301 study (68) was a global, multicenter, open-label phase III randomized trial involving 250 patients with recurrent or metastatic cervical cancer. All patients received tisotumab vedotin at 2 mg/kg q3w. Ocular AEs were observed in 52.8% of patients, with a median time to onset of 1.22 months. Conjunctivitis was reported in 31.2%, keratitis in 15.6%, and additional AEs included dry eye, blepharitis and eye discharge. Grade 3 or higher ocular toxicities included keratitis in 2%, punctate keratitis in 0.4%, and cataract in 0.4% of patients.

A multicenter retrospective study (69) evaluated ocular toxicities in five patients treated with tisotumab vedotin. All patients developed conjunctival subepithelial fibrosis; two experienced pseudomembranous conjunctivitis, of whom one subsequently developed conjunctival scarring. One patients developed infectious keratitis and was treated with corneal transplantation. A pharmacovigilance analysis of the FAERS database (38) revealed that, in real-world settings, ocular AEs associated with tisotumab vedotin primarily included dry eye, keratitis, ulcerative keratitis, punctate keratitis, ocular hyperaemia, among others.

#### 4.2.4 Brentuximab vedotin

Brentuximab vedotin consists of a humanized IgG1 monoclonal antibody targeting CD30, MMAE, and a cleavable valine-citrulline dipeptide linker. No ocular toxicities have been reported in published clinical trials of brentuximab vedotin. However, ocular AEs such as uveitis (70), panuveitis (71), Vogt-Koyanagi-Harada-like granulomatous pan uveitis (72) and purtscher-like retinopathy (73) have been described in case reports. A pharmacovigilance analysis based on the FAERS database (38) identified ocular AEs associated with brentuximab vedotin, including blindness, blindness unilateral, papilledema, retinal haemorrhage and visual acuity reduced transiently. Notably, strong signals were detected for visual acuity reduced transiently and papilledema. In addition, a signal detection study using the Japanese Adverse Drug Event Report database identified an association between brentuximab vedotin and retinal detachment (74). In real-world settings, the median time to onset of ocular toxicity was 36 days (49).

#### 4.2.5 Telisotuzumab vedotin

Telisotuzumab vedotin is an ADC targeting c-mesenchymal-epithelial transition factor, composed of a humanized IgG1 monoclonal antibody, a cleavable valine-citrulline dipeptide linker, and MMAE. The phase II LUMINOSITY trial (75) evaluated the efficacy and safety of telisotuzumab vedotin at a dose of 1.9 mg/kg every two weeks (q2w) in patients with locally advanced or metastatic nonsquamous non-small cell lung cancer (NSCLC). According to the FDA risk assessment summary of the LUMINOSITY trial, ocular AEs occurred in 25% of patients, with a median time to onset of 47 days. The most frequently observed ocular toxicities were blurred vision in 15%t of patients, keratitis in 11%, and dry eye in 5%. Severe ocular AEs, defined as grade 3 or higher, included blurred vision in 1.2% and keratitis in 0.6% of patients (76). No published literature reports of ocular AEs associated with telisotuzumab vedotin have been identified in the postmarketing setting.

#### 4.2.6 Disitamab vedotin

Disitamab vedotin consists of a humanized anti-HER2 monoclonal antibody, a cleavable maleimidocaproyl-valyl-citrullinyl-p-aminobenzyloxycarbonyl linker and MMAE. A pooled safety analysis of six clinical trials (C001, C002, C005, C006, C008, and C009) involving disitamab vedotin included 394 patients who received either 2 mg/kg q2w for urothelial cancer and breast cancer or 2.5 mg/kg q2w for gastric cancer. Reported ocular AEs included increased lacrimation, blurred vision, and periorbital swelling, with incidences ranging from 0.1% to 1% (77). No published literature reports of ocular AEs associated with disitamab vedotin have been identified in the postmarketing setting.

#### 4.2.7 Belantamab mafodotin

Belantamab mafodotin is an ADC targeting B-cell maturation antigen. It comprises MMAF, conjugated to the humanized monoclonal antibody via a non-cleavable maleimidocaproyl linker. DREAMM-1 trial (78) evaluated belantamab mafodotin in 73 patients with relapsed or refractory multiple myeloma(RRMM). In the dose-escalation phase, patients received belantamab mafodotin at doses ranging from 0.03 mg/kg to 4.6 mg/kg q3w, with ocular AEs reported in 53% of the 38 participants. In the dose-expansion cohort, patients were treated at 3.4 mg/kg q3w, and 63% of the 35 participants experienced ocular AEs. The most commonly reported ocular AEs were corneal events included but were not limited to blurred vision, dry eye, photophobia, and others. Grade 3 or higher corneal events comprised keratitis in 6% of patients, dry eye, eye pain, and eye disorders each in 3%, as well as retinal detachment also in 3%. The median time to onset of corneal events was 23 days and the median duration for patients with a resolution date was 30 days. In DREAMM-2 trial (79), 194 patients with multiple myeloma received belantamab mafodotin at a dose of either 2.5 mg/kg q3w (n=95) or 3.4 mg/kg q3w (n=99). Corneal events emerged as the most common AEs, occurring in up to 73% of patients. In the 2.5 mg/kg group, grade 1–2 corneal events occurred in 43% of patients and grade 3 in 27%, with no grade 4 events reported. In the 3.4 mg/kg group, grade 1–2 events were observed in 54%, grade 3 in 20%, and grade 4 in 1%. Four patients (one in the 2.5 mg/kg group and three in the 3.4 mg/kg group) permanently discontinued treatment due to keratopathy, which represented the most common cause of permanent discontinuation. Keratopathy was also the leading reason for dose reductions and treatment delays or interruptions. Other ocular AEs included blurred vision (22% in the 2.5 mg/kg group and 30% in the 3.4 mg/kg group) and dry eye (14% and 23%, respectively). Most ocular AEs were mild to moderate, though some required dose adjustments or treatment interruptions. In DREAMM-3 study (80), 66% of the 217 patients with RRMM treated with belantamab mafodotin at 2.5mg/kg q3w experienced ocular AEs of special interest, with 29% reporting AEs of grade 3 or higher. The most commonly reported AEs included blurred vision and visual acuity reduced. The median time to onset of ocular toxicity was 40 days, with a median duration of 66 days.

Several real-world studies (49, 81–88) have reported ocular toxicity rates ranging from 44% to 89.6% in patients treated with belantamab mafodotin. The median time to onset was 39 days, with keratopathy and vision loss being the most frequently observed symptoms. These findings showed a safety profile of belantamab mafodotin consistent with the results in the registrational trials. In a retrospective analysis (81) involving 106 patients with RRMM treated with belantamab mafodotin, 89.6% experienced ocular AEs. Among them, approximately 68.4% developed keratopathy and 36.8% reported blurred vision. Grade 2 and grade 3 keratopathy occurred in 16.8% and 38.9% of patients, respectively. In another study (82), 56 patients with RRMM were treated with belantamab mafodotin. Within four months of treatment, 71.4% experienced new or worsening keratopathy, with grade 3 AEs observed in 54% of patients. Keratopathy led to treatment interruption in 48% of patients. Permanent discontinuation in 25% due to ocular toxicity. Dileo et al (83) reported real-world clinical outcomes from 81 patients with RRMM treated with belantamab mafodotin. Ocular toxicity of any grade occurred in 69% of patients, while grade 3 or higher ocular AEs were observed in 43%. The median time to onset of ocular AEs was 21 days, and the median time to resolution to grade 1 or complete recovery was 42 days. Keratopathy were reported in 69% of patients, with grade 3 or higher keratopathy occurring in 30%. Additionally, 32% of patients experienced a visual acuity reduced to below 20/50 on the visual acuity scale. A case report (89) described a 60-year-old male patient with multiple myeloma who developed epithelial crystal-like deposits in the cornea and experienced a visual acuity reduced following three doses of belantamab mafodotin at 2.5 mg/kg. Symptoms improved following discontinuation of treatment and administration of artificial tears and topical loteprednol etobonate eye drops. Belantamab mafodotin was subsequently resumed at a reduced dose of 1.9 mg/kg without worsening of ocular toxicity. Another case report (90) described a 61-year-old female patient with multiple myeloma who developed blurred vision and microcystic keratopathy after six weeks treatment of belantamab mafodotin at a dose of 2.5 mg/kg. Ocular symptoms resolved following treatment interruption, and therapy was successfully resumed at a reduced dose of 1.9 mg/kg.

### 4.3 Pyrrolobenzodiazepine

#### 4.3.1 Loncastuximab tesirine

Loncastuximab tesirine is composed of a humanized IgG1 monoclonal antibody targeting CD19, conjugated to pyrrolobenzodiazepine via a cleavable valine-alanine dipeptide linker. In LOTIS-2 trial (91), 145 patients with relapsed or refractory diffuse large B-cell lymphoma received loncastuximab tesirine at 150 µg/kg by intravenous infusion q3w, with the dose reduced to 75 µg/kg q3w after two cycles. One patient developed grade 3 cataract. No published literature reports of ocular AEs associated with loncastuximab tesirine have been identified in the postmarketing setting.

### 4.4 Calicheamicin

#### 4.4.1 Gemtuzumab ozogamicin

Gemtuzumab ozogamicin is composed of a CD33-directed immunoglobulin G4 (IgG4) monoclonal antibody linked to calicheamicin via a cleavable acid-labile hydrazone linker, used 4-(4′-acetylphenoxy)butanoic acid hydrazide. According to the FDA medical review (92), ocular AEs occurred in approximately 8% of patients, with severe AEs reported in 1%. These events were predominantly hemorrhagic or infusion-related, included eye hemorrhage, retinal hemorrhage, conjunctival hemorrhage, scleral hemorrhage, and eyelid hematoma, each occurring in approximately 1% of patients. Eye swelling and periorbital edema were less frequent, each reported in about 0.4% of cases. Grade 3 retinal hemorrhage occurred in 0.4% of patients. A study involving nine patients also described one instance of grade 4 ocular bleeding, attributed to gemtuzumab ozogamicin induced thrombocytopenia (93). In a pharmacovigilance analysis (38), twelve ocular AE reports of gemtuzumab ozogamicin were included. Gemtuzumab ozogamicin demonstrated a weak signal for ocular toxicity. Except for retinal hemorrhage, which showed a marginal signal (IC025 = 0.08), all other ocular AEs exhibited IC025 value below zero, indicating no significant association.

#### 4.4.2 Inotuzumab ozogamicin

Inotuzumab ozogamicin consists of a humanized IgG4 monoclonal antibody targeting CD22, conjugated to calicheamicin via a cleavable acid-labile hydrazone linker, specifically 4-(4′-acetylphenoxy)butanoic acid hydrazide. In the phase III INO-VATE ALL trial (94, 95), 164 patients with relapsed or refractory B-cell acute lymphoblastic leukemia received standard-dose inotuzumab ozogamicin. Reported ocular toxicities included conjunctival hemorrhage and eyelid bleeding. however, these events were categorized under vascular disorders. A pharmacovigilance study (38) analyzed 10 ocular AE reports of inotuzumab ozogamicin, predominantly from pediatric patients. The ocular toxicities associated with inotuzumab ozogamicin were conjunctival haemorrhage and eyelid oedema.

### 4.5 Topoisomerase I inhibitor

#### 4.5.1 Trastuzumab deruxtecan

Trastuzumab deruxtecan (T-DXd) is an ADC composed of trastuzumab linked to DXd via a cleavable tetrapeptide linker, glycine-glycine-phenylalanine-glycine. In DESTINY-Breast01 trial (96), which included 253 HER2-positive MBC patients treated with T-DXd at doses of 5.4mg/kg, 6.4mg/kg, or 7.4 mg/kg q3w, dry eye was reported in approximately 11.5% of patients, including one case of grade 4. In DESTINY-Breast02 trial (97, 98), among 404 patients with unresectable or metastatic HER2-positive breast cancer treated with T-DXd 5.4 mg/kg q3w, 6% experienced dry eye and 3% reported blurred vision. Similarly, in DESTINY-Breast03 trial (98, 99), blurred vision occurred in 3.5% of 257 patients with HER2-positive MBC receiving the same regimen. The DESTINY-Breast04 study (98, 100) reported a 4.9% incidence of blurred vision among 371 patients with HER2-low MBC treated with T-DXd at 5.4 mg/kg q3w. Cases of eye hemorrhage were also observed. In DESTINY-Breast06 trial (98, 101), involving 434 patients with hormone receptor (HR)-positive MBC with low or ultralow HER2 expression, dry eye was reported in 7% and blurred vision in 5%. Periorbital edema and eyelid edema have also been documented across four studies (96, 102–104). According to pharmacovigilance researches from the FAERS database (38, 105, 106), reported ocular AEs associated with T-DXd include excessive eye blinking, keratitis, corneal disorder, punctate keratitis, eye hematoma and pinguecula. In real-world settings, the median time to onset of T-DXd related ocular toxicity was approximately 91.5 days (41).

#### 4.5.2 Sacituzumab govitecan

Sacituzumab govitecan is composed of a monoclonal antibody targeting Trop-2 linked to SN-38 via a cleavable, pH-sensitive CL2A linker. In IMMU-132–01 trial (107, 108), which enrolled 108 patients with metastatic triple-negative breast cancer (TNBC) receiving sacituzumab govitecan at 10 mg/kg on days 1 and 8 q3w, cases of periorbital edema was reported, but these were counted as part of edema related AEs. In ASSENT study (109), among 258 patients with relapsed or refractory metastatic TNBC treated with sacituzumab govitecan at 10 mg/kg on days 1 and 8 q3w, ocular AEs were reported in 5% of patients, all of which were grade 1. Postmarketing studies (38, 110–112) of the FAERS database have identified ocular AEs potentially associated with sacituzumab govitecan, including cataract, eyelid ptosis, periorbital edema, dry eyes, ulcerative keratitis, increased lacrimation, visual impairment, among others. In real-world settings, the median time to onset of ocular toxicity was 21 days (49).

#### 4.5.3 Datopotamab deruxtecan

Datopotamab deruxtecan is a Trop-2 targeting ADC composed of a monoclonal antibody linked to DXd via a cleavable tetrapeptide linker, glycine-glycine-phenylalanine-glycine. In TROPION-PanTumor01 trial (113, 114), among patients with NSCLC cancer enrolled in the phase I dose-expansion cohort, datopotamab deruxtecan was administered at 4 mg/kg, 6 mg/kg, or 8 mg/kg q3w to a total of 180 patients. Ocular surface toxicities were reported in 32.2% of patients, with grade 3 AEs occurring in 1.7%. The most commonly observed ocular surface toxicities were dry eye, lacrimation increased and blepharitis, while grade 3 ocular surface toxicities included keratitis and ulcerative keratitis. In the breast cancer cohort, all patients received datopotamab deruxtecan at 6 mg/kg q3w. Among 41 patients with HR+/HER2- breast cancer, ocular surface toxicities occurred in 41.5%, and among 44 patients with TNBC, the incidence was 36.4%, all of which were grade 1 or 2. The most frequently reported ocular surface toxicities included dry eye (24.4% in HR+/HER2- and 15.9% in TNBC) and keratitis (9.8% in HR+/HER2- and 2.3% in TNBC). One patient developed treatment-related cataract. In TROPION-Breast01 trial (115), 360 patients with previously treated, unresectable or metastatic HR+/HER2- breast cancer received datopotamab deruxtecan at 6 mg/kg q3w. Ocular AEs occurred in 40% of patients, the majority of which were grade 1, including dry eye, keratitis, blepharitis, increased lacrimation, meibomian gland dysfunction, and others. Grade 3 ocular AEs were reported in 0.6% of patients, comprising two cases each of dry eye and keratitis. The median time to onset of ocular toxicity was 2.1 months. No published literature reports of ocular AEs associated with datopotamab deruxtecan have been identified in the postmarketing setting.

#### 4.5.4 Sacituzumab tirumotecan

Sacituzumab tirumotecan is an ADC composed of a targeting Trop-2 monoclonal antibody, a belotecan-derived topoisomerase I inhibitor (KL610023), and a non-cleavable sulfonyl pyrimidine-CL2A-carbonate linker. In the phase IIdose-expansion cohort of the KL264–01 trial (116), one case of dry eye leading to treatment discontinuation was reported among 36 patients with TNBC treated with sacituzumab tirumotecan at 5 mg/kg. In part II of the OptiTROP-Lung03 trial (117), 91 patients with locally advanced or metastatic EGFR-mutant NSCLC received sacituzumab tirumotecan at 5 mg/kg on days 1 and 15 every 4 weeks. Ocular AEs were reported in 2% of patients: one case of blurred vision and one of ophthalmodynia, both grade 1. No grade 3 or higher ocular toxicities occurred. In OptiTROP-Breast01 trial (118), among 130 patients with locally recurrent or metastatic TNBC receiving sacituzumab tirumotecan at 5 mg/kg q2w, xerophthalmia occurred in 2.3% of patients, all grade 1or 2, and blurred vision in 1.5%, all grade 1. No grade 3 or higher ocular AEs were observed. No published literature reports of ocular AEs associated with sacituzumab tirumotecan have been identified in the postmarketing setting.

#### 4.5.5 Trastuzumab rezetecan

Trastuzumab rezetecan is a HER2-targeting ADC composed of trastuzumab, a cleavable glycine-glycine-phenylalanine-glycine tetrapeptide linker and the topoisomerase I inhibitor payload SHR169265. The NCT04446260 trial (119), a global multicenter phase I study, reported one case of grade 3 or higher ulcerative keratitis among 30 non-Asian patients with advanced solid tumors harboring HER2 expression or mutation who received trastuzumab rezetecan at a dose of 4.8 mg/kg. The Chinese prescribing information for trastuzumab rezetecan (120) includes a pooled safety analysis from three clinical trials, identifying blurred vision as a common AE, while conjunctival hyperemia and lacrimation increased were infrequent. No published literature reports of ocular AEs associated with trastuzumab rezetecan have been identified in the postmarketing setting.

### 4.6 Pseudomonas exotoxin A

#### 4.6.1 Moxetumomab pasudotox

Moxetumomab pasudotox is a recombinant immunotoxin composed of the variable fragment of an anti-CD22 monoclonal antibody genetically fused to the truncated Pseudomonas exotoxin A. This engineered construct does not utilize a traditional chemical linker; instead, the antibody and payload are directly fused at the genetic level. In NCT01829711 trial (121, 122), a total of 80 patients with relapsed or refractory hairy cell leukemia received moxetumomab pasudotox at a dose of 40μg/kg on days 1, 3, and 5 of a 28-day cycle. Common ocular AEs included blurred vision in 9% of patients, dry eye in 8%, cataract in 5%, ocular discomfort/pain in 4%, ocular swelling/periorbital edema in 4%, and others. No published literature reports of ocular AEs associated with moxetumomab pasudotox have been identified in the postmarketing setting.

### 4.7 IRDye700DX

#### 4.7.1 Cetuximab saratolacan

Cetuximab saratolacan is an ADC composed of cetuximab covalently linked to the photosensitizer IRDye700DX, representing a novel type of photoimmunotherapy agents. In the phase I/IIa (NCT02422979) trial (123), cetuximab saratolacan was administered at a dose of 640 mg/m² during the phase IIa. The light dose was fixed at 50 J/cm² for superficial tumors and 100 J/cm fiber diffuser length for interstitial tumors. Among 30 patients with locally recurrent or refractory head and neck squamous cell carcinoma, eye disorders occurred in 6.7% of patients, including one patient who experienced grade 3 or higher periorbital edema. No published literature reports of ocular AEs associated with cetuximab saratolacan have been identified in the postmarketing setting.

## 5 Management of ocular toxicity

Currently, no standardized guidelines exist for the prevention and management of ADC-associated ocular toxicity. Nonetheless, well-designed ocular care plans and appropriate dose modifications can substantially reduce both the frequency and severity of ocular AEs. Most ocular AEs induced by ADCs are reversible with early detection and discontinuation of treatment. Preventive strategies include the use of preservative-free artificial tears or lubricating eye drops throughout treatment, administration of topical corticosteroid drops before and during therapy, such as dexamethasone 0.1%, prednisolone 1% or fluorometholone 0.1%, and instillation of vasoconstrictor eye drops prior to each ADC infusion (124). brimonidine tartrate 0.2% has been demonstrated to be effective in alleviating infusion-related conjunctival hyperemia. It is recommended to instill three drops prior to each ADCs administration to achieve vasoconstriction and reduce superficial hyperperfusion (125). Using cooling eye pads during each administration may also be beneficial. Patients should avoid wearing contact lenses during treatment unless explicitly advised by an ophthalmologist. Baseline ophthalmologic assessments, including visual acuity testing and slit-lamp examination, are recommended prior to initiation of belantamab mafodotin, tisotumab vedotin and mirvetuximab soravtansine. Regular assessments during therapy are also advised to detect early signs of ocular toxicity. Clinicians should monitor for conjunctivitis and dry eye symptoms, including periorbital redness, pain, and irritation, and remain alert to changes in vision. Patients should be encouraged to report any new or worsening ocular symptoms promptly. In the event of sudden vision loss or eye pain accompanied by photophobia, immediate ophthalmologic evaluation is warranted. Patients should also be educated to maintain proper periocular hygiene, including regular cleansing of the eyelid margins with sterile saline to reduce the risk of infection.

Dose reduction and treatment interruption serve as important strategies to mitigate ocular AEs. If ocular AEs occur, treatment modifications may be considered: continuing treatment at the original dose with preservative-free artificial tears for grade 1, pausing therapy and resuming at one dose level lower after resolution of grade 2 or 3, and permanently discontinuing the ADC for grade 4 (124). Treatment of dry eye should be initiated when symptoms reach grade 2 or higher, or if grade 1 symptoms persist for more than one week. Preservative-free artificial tears should be used preferentially. Additional therapies may be added according to severity, including high molecular weight lubricants, warm compresses, immunomodulators such as cyclosporine 0.05%, and short term corticosteroid therapy. Mild keratopathy can be managed with high viscosity artificial tears or ophthalmic gels. Moderate to severe keratopathy require prophylactic antibiotic eye drops, such as tobramycin or moxifloxacin, and in some patients, short term combination with corticosteroid drops under the supervision of an ophthalmologist. For mild conjunctivitis, artificial tears are recommended initially. If inflammation is pronounced, short-term corticosteroid eye drops, such as fluorometholone 0.1% administered 2–4 times daily, may be added. In cases with ocular discharge, antibiotic eye drops can be used concurrently (126). Notably, it is important to distinguish between the presence of keratopathy and its actual impact on visual function. Some patients may exhibit significant keratopathy without noticeable symptoms, whereas others may experience blurred vision that meaningfully impairs their quality of life. The DREAMM-2 study demonstrated that keratopathy often precedes or accompanies the onset of blurred vision, and even low-grade keratopathy can significantly impair visual function (26, 79). Many elderly patients have pre-existing ocular conditions such as cataracts. Optimizing visual function before initiating treatment, for example by recommending cataract surgery when appropriate, may help prevent further visual deterioration and improve quality of life. Moreover, enhancing patient education on recognizing ocular symptoms such as blurred vision can facilitate timely treatment adjustment. Proactive monitoring and dose management allow clinicians to minimize AEs and improve tolerability.

Given the inflammatory component of ADC-associated ocular toxicity (127), prophylactic use of corticosteroid eye drops has become an part of ocular toxicity management. In InnovaTV 201 study (64), implementation of a preventive regimen, including topical dexamethasone 0.1% eye drops, led to a marked reduction in the incidence of conjunctivitis associated with tisotumab vedotin. As a result, topical corticosteroid eye drops, administered as one drop per eye three times daily for 72 hours after each infusion, have become the standard premedication regimen recommended in the prescribing information for tisotumab vedotin. However, in DREAMM-2 study (79), prophylactic corticosteroid eye drops failed to prevent epithelial changes of the cornea induced by belantamab mafodotin. Although the daily prophylactic use of corticosteroid eye drops in patients receiving mirvetuximab soravtansine does not reduce the overall incidence of ocular AEs, it can help decrease the need for dose modifications due to ocular toxicity (128). It is important to note that prolonged use of topical corticosteroids eye drops carries the risk of ocular infection and elevated intraocular pressure (129). For other ADCs, corticosteroid eye drops prophylaxis has not been formally evaluated, and its efficacy remains unknown. Routine ophthalmologic examinations are critical during ADC treatment to monitor for ocular toxicity. Recommended assessments include best-corrected visual acuity, slit-lamp microscopy, optical coherence tomography (OCT), and confocal microscopy (125, 127, 130, 131). Microcyst-like epithelial changes induced by ADC can be identified using slit-lamp microscopy, OCT, and confocal microscopy (26, 127). Oncologists should carefully review patients’ ophthalmologic examination outcomes and maintain ongoing communication with ophthalmologist to determine whether dose modifications or treatment discontinuation are necessary. The oncology care team should also guide patients in managing the influence of ocular toxicity on daily activities. This multidisciplinary treatment strategy helps patients better navigate complex therapies and enhances adherence.

## 6 Outlook

Ocular toxicity is a common adverse effect of ADCs according to available data. As summarized in **Table 1**, ocular toxicities associated with ADCs manifest in diverse forms, involving the ocular surface, intraocular structures, and ocular adnexa. Ocular surface disorders, particularly dry eye, conjunctivitis and increased lacrimation, frequently occur and are generally mild to moderate in severity. Corneal toxicity, often described as keratopathy or microcyst-like epithelial changes, is one of the most distinctive ocular AEs associated with ADCs, especially for agents carrying microtubule inhibitor payloads such as MMAF or DM4. Blurred vision, visual acuity reduced and photophobia represent the most frequently reported subjective visual symptoms associated with ADCs, and in severe cases, may necessitate dose modification or treatment interruption. Posterior segment toxicities are relatively rare but may have greater potential impact on visual function. Reported manifestations include age-related macular degeneration, choroidal neovascularisation, retinal detachment and papilledema of optic nerve. However, a direct causal relationship between these AEs and ADCs has not been clearly established. Ocular hemorrhage, periorbital edema, cataract and eyelid-related AEs have also been observed with some ADCs. Most dose-limiting toxicities associated with ADCs are not the result of specific targeting of normal tissues (132). Regardless of the antigen expression level in normal tissues, ADCs sharing the same type of payload often exhibit highly consistent profile of grade 3 or 4 toxicity in clinical settings (133). Previous studies have reported an overall ocular toxicity incidence of 10.45% with ADCs, most commonly observed in those carrying MMAF, MMAE, or DM4 as payload (133, 134).

Our study indicates that ocular toxicity can occur with all currently used payload types in ADCs. Severe ocular AEs have been reported in ADCs carrying MMAE, MMAF, DM4, topoisomerase I inhibitors, IRDye700DX, and calicheamicin. Ocular toxicity associated with ADCs does not originate solely from the payload. Ocular AEs have been observed across different payload types, and even among ADCs sharing the same payload, the incidence and severity of ocular toxicity can vary substantially. One study identified linker type as the only factor significantly associated with ocular toxicity. In particular, non-cleavable linkers were strongly correlated with an increased risk of ocular AEs, independent of the ADC target, antibody, payload, or drug-to-antibody ratio (DAR) (135). Non-cleavable linkers, particularly the maleimidocaproyl linker used in belantamab mafodotin, are designed to provide greater plasma stability. However, it may undergo retro-Michael reactions under physiological conditions, leading to premature payload release and increased ocular toxicity. Clarifying the interplay between linkers, payloads, and local tissue biology is crucial for minimizing ocular off-target toxicity in the development of innovative ADCs. The use of self-hydrolyzing maleimide-based linkers may represent a promising approach. These linkers enhance the stability of ADCs in circulation through controlled hydrolysis while minimizing premature payload release, thereby potentially reducing the risk of ocular toxicity (136).

Furthermore, ADC hydrophobicity often correlates with DAR, and as DAR increases, the number of payload molecules per antibody rises, narrowing the therapeutic window and reducing tolerability (15). Highly hydrophobic payloads such as MMAF tend to accumulate in non-target tissues, posing a particular risk to sensitive sites like the eye. As such, rational optimization of payload characteristics is crucial to mitigating DAR-associated toxicities. At present, linker design remains highly constrained, with most approved ADCs relying on a limited set of established linker chemistries. In the future, computational technologies may accelerate the development of innovative linker for ADCs. By combining molecular dynamics simulations with machine learning, researchers can generate and evaluate large combinatorial libraries of linker types, conjugation sites, and DAR that are experimentally accessible (137). Artificial intelligence models trained through transfer learning and reinforcement learning on large-scale molecular datasets can leverage self-attention mechanisms to generate novel linkers with high structural diversity and synthetic feasibility (138). These approaches not only facilitate the identification of optimal linker types and improve the design efficiency of ADCs by minimizing experimental trial and error, but also expand the chemical space for linker design, offering new avenues for the discovery and optimization of novel ADCs.

## 7 Conclusions

Despite their substantial technological advantages and broad clinical potential across a range of solid and hematologic malignancies, ADCs remain constrained by toxicity, which continues to limit therapeutic window expansion. Ocular AEs have been reported for all approved ADCs, both in clinical trials and postmarketing settings. This widespread toxicity appears to be only weakly correlated with any specific payload type. With appropriate toxicity management strategies and monitoring plans, ocular toxicity can often be effectively controlled. Multidisciplinary collaboration plays a critical role in the early detection, treatment, and surveillance of ocular toxicity. Incorporating emerging technologies and novel design strategies into linker innovation during drug development may help reduce ocular toxicity and optimize the benefit-risk profile of ADCs.
