# Supplementary material for: Ocular toxicity associated with antibody-drug conjugates in cancer therapy: a comprehensive review
Source: Front Immunol. 2026 Apr 27;17:1698458. doi: 10.3389/fimmu.2026.1698458 (PMC13158211; doi:10.3389/fimmu.2026.1698458)
Supplement: Supplementary file 1 [file Table1.docx]

**Supplementary Material 1**

Supplementary Table 1 Search strategy.

| **Pubmed** | |
| --- | --- |
| #1 | Ado-Trastuzumab emtansine [Title/Abstract] or Trastuzumab Emtansine [Title/Abstract] or Trastuzumab DM1 [Title/Abstract] or T-DM1 [Title/Abstract] or huN901DM1 [Title/Abstract] or Mirvetuximab soravtansine [Title/Abstract] or IMGN853 [Title/Abstract] or Polatuzumab vedotin [Title/Abstract] or DCDS4501A [Title/Abstract] or RG7596 [Title/Abstract] or RO5541077-000 [Title/Abstract] or ACD79B-VCMMAE [Title/Abstract] or FCU2711 [Title/Abstract] or Enfortumab vedotin [Title/Abstract] or ASG-22CE [Title/Abstract] or AGS-22M6E [Title/Abstract] or Tisotumab vedotin [Title/Abstract] or Brentuximab vedotin [Title/Abstract] or SGN35 [Title/Abstract] or cAC10vcMMAE [Title/Abstract] or Telisotuzumab vedotin [Title/Abstract] or ABBV-399 [Title/Abstract] or Disitamab vedotin [Title/Abstract] or RC48 [Title/Abstract] or Belantamab mafodotin [Title/Abstract] or GSK2857916 [Title/Abstract] or Loncastuximab tesirine [Title/Abstract] or ADCT-402 [Title/Abstract] or Gemtuzumab [Title/Abstract] or Gemtuzumab ozogamicin [Title/Abstract] or CMA 676 [Title/Abstract] or Inotuzumab ozogamicin [Title/Abstract] or CMC 544 [Title/Abstract] or Trastuzumab deruxtecan [Title/Abstract] or DS-8201 [Title/Abstract] or Sacituzumab govitecan [Title/Abstract] or IMMU-132 [Title/Abstract] or HRS7-SN38 [Title/Abstract] or Datopotamab deruxtecan [Title/Abstract] or DS-1062a [Title/Abstract] or Dato-DXd [Title/Abstract] or Sacituzumab tirumotecan [Title/Abstract] or SKB264 [Title/Abstract] or Trastuzumab rezetecan [Title/Abstract] or SHR-A1811 [Title/Abstract] or Moxetumomab pasudotox [Title/Abstract] or CAT8015 [Title/Abstract] or Cetuximab saratolacan [Title/Abstract] or RM-1929 [Title/Abstract] |
| #2 | Trastuzumab emtansine [Title/Abstract] or Kadcyla [Title/Abstract] or Mirvetuximab soravtansine [Title/Abstract] or Elahere [Title/Abstract] or Polatuzumab vedotin [Title/Abstract] or Polivy [Title/Abstract] or Enfortumab vedotin [Title/Abstract] or Padcev [Title/Abstract] or Tisotumab vedotin [Title/Abstract] or Tivdak [Title/Abstract] or Brentuximab vedotin [Title/Abstract] or Adcetris [Title/Abstract] or Telisotuzumab vedotin [Title/Abstract] or Emrelis [Title/Abstract] or Disitamab vedotin [Title/Abstract] or Aidixi [Title/Abstract] or Belantamab mafodotin [Title/Abstract] or Blenrep [Title/Abstract] or Loncastuximab tesirine [Title/Abstract] or Zynlonta [Title/Abstract] or Gemtuzumab ozogamicin [Title/Abstract] or Mylotarg [Title/Abstract] or Inotuzumab ozogamicin [Title/Abstract] or Besponsa [Title/Abstract] or Trastuzumab deruxtecan [Title/Abstract] or Enhertu [Title/Abstract] or Sacituzumab govitecan [Title/Abstract] or Trodelvy [Title/Abstract] or Datopotamab deruxtecan [Title/Abstract] or Datroway [Title/Abstract] or Sacituzumab tirumotecan [Title/Abstract] or Trastuzumab rezetecan [Title/Abstract] or Moxetumomab pasudotox [Title/Abstract] or Lumoxiti [Title/Abstract] or Cetuximab saratolacan [Title/Abstract] |
| #3 | randomized controlled trial [pt] or clinical trial [pt] |
| #4 | eye [Title/Abstract] or ocular [Title/Abstract] or ophthalm* [Title/Abstract] |
| #5 | case report [Title/Abstract] or case series [Title/Abstract] or real world [Title/Abstract] or FAERS [Title/Abstract] |
| #6 | #1 and #3 |
| #7 | #2 and #4 and #5 |
| **Cochrane Central Register of Controlled Trials** | |
| #1 | (Ado-Trastuzumab emtansine or Trastuzumab Emtansine or Trastuzumab DM1 or T-DM1 or huN901DM1):ti,ab,kw |
| #2 | (Mirvetuximab soravtansine or IMGN853):ti,ab,kw |
| #3 | (Polatuzumab vedotin or DCDS4501A or RG7596 or RO5541077-000 or ACD79B-VCMMAE or FCU2711):ti,ab,kw |
| #4 | (Enfortumab vedotin or ASG-22CE or AGS-22MSE or AGS-22M6E or AGS-22ME):ti,ab,kw |
| #5 | Tisotumab vedotin:ti,ab,kw |
| #6 | (Brentuximab vedotin or SGN35 or CAC101006 or cAC10vcMMAE):ti,ab,kw |
| #7 | (Telisotuzumab vedotin or ABBV-399 or ABT-399 or PR-1420682 or ABT-700-VCMMAE):ti,ab,kw |
| #8 | (Disitamab vedotin or RC48):ti,ab,kw |
| #9 | (Belantamab mafodotin or GSK2857916):ti,ab,kw |
| #10 | (Loncastuximab tesirine or ADCT-402):ti,ab,kw |
| #11 | (Gemtuzumab or Gemtuzumab ozogamicin or CMA 676):ti,ab,kw |
| #12 | (Inotuzumab ozogamicin or CMC 544):ti,ab,kw |
| #13 | (Trastuzumab deruxtecan or DS-8201):ti,ab,kw |
| #14 | (Sacituzumab govitecan or IMMU-132 or HRS7-SN38):ti,ab,kw |
| #15 | (Datopotamab deruxtecan or DS-1062a or Dato-DXd):ti,ab,kw |
| #16 | (Sacituzumab tirumotecan or SKB264):ti,ab,kw |
| #17 | (Trastuzumab rezetecan or SHR-A1811):ti,ab,kw |
| #18 | (Moxetumomab pasudotox or CAT8015):ti,ab,kw |
| #19 | (Cetuximab saratolacan or RM-1929):ti,ab,kw |
| #20 | or/#1-#19 |
