# Supplementary material for: Ocular toxicity associated with antibody-drug conjugates in cancer therapy: a comprehensive review
Source: Front Immunol. 2026 Apr 27;17:1698458. doi: 10.3389/fimmu.2026.1698458 (PMC13158211; doi:10.3389/fimmu.2026.1698458)
Supplement: Supplementary file 2 [file Table2.docx]

**Supplementary Material 2**

Supplementary Table 2 Ocular AEs associated with ADCs in clinical trials.

| ADC | Ocular AEs in any grade | Ocular AEs ≥grade 3 | Reference |
| --- | --- | --- | --- |
| Ado-Trastuzumab emtansine | TDM3569g:dry eye(10.7%), cataract, ocular surface disease, punctuate keratitis | TDM3569g:cataract, ocular surface disease, punctuate keratitis | [32] |
|  | TDM4258g:dry eye, increased lacrimation, blurred vision/visual impairment, conjunctivitis | / | [33] |
|  | EMILIA:blurred vision(4.5%), conjunctivitis(3.9%), dry eye(3.9%),increased lacrimation(3.3%) | / | [34], [35] |
|  | KATHERINE:blurred vision(3.9%), conjunctivitis(3.5%), dry eye(4.5%), increased lacrimation(6%) | / | [35], [37] |
| Mirvetuximab soravtansine | SORAYA:visual impairment(43%), keratopathy(36%)[including corneal cyst, corneal disorder, corneal epithelial microcysts, keratitis, keratopathy, limbal stem-cell deficiency, corneal opacity, corneal erosion, corneal pigmentation, corneal deposits, keratitis interstitial, punctate keratitis, and corneal epithelial defect], dry eye(25%), cataract(10%), photophobia(14%), blurred vision(41%) | SORAYA:visual impairment(6%), keratopathy(9%)[including corneal cyst, corneal disorder, corneal epithelial microcysts, keratitis, keratopathy, limbal stem-cell deficiency, corneal opacity, corneal erosion, corneal pigmentation, corneal deposits, keratitis interstitial, punctate keratitis, and corneal epithelial defect], dry eye(2%), blurred vision(6%) | [44], [45] |
|  | FORWARD I:blurred vision(42%), keratopathy(32.5%)[including keratopathy, keratitis, punctate keratitis, corneal epithelial microcysts, corneal cyst, corneal deposits, limbal stem cell deficiency, corneal disorder, corneal opacity, corneal erosion, corneal pigmentation, keratitis interstitial, and corneal epithelium defect], dry eye(25.9%), visual acuity reduced(19.3%) | FORWARD I:blurred vision(2.5%), keratopathy(1.2%)[including keratopathy, keratitis, punctate keratitis, corneal epithelial microcysts, corneal cyst, corneal deposits, limbal stem cell deficiency, corneal disorder, corneal opacity, corneal erosion, corneal pigmentation, keratitis interstitial, and corneal epithelium defect], dry eye(1.2%) | [46] |
|  | MIRASOL:blurred vision(40.8%), keratopathy(32.1%), dry eye(28%), photophobia(17.9%), cataract(14.7%), visual acuity reduced(11.9%), eye pain(9.2%), keratitis(5%), vitreous floaters(5%) | MIRASOL:blurred vision(7.8%), keratopathy(9.2%), dry eye(3.2%), photophobia(0.5%), cataract(3.2%), visual acuity reduced(3.2%), keratitis(1.4%) | [47] |
| Polatuzumab vedotin | GO29365:visual acuity reduced(1.2%) | / | [52] |
|  | Dry eye, blurred/impaired vision | / | [53] |
| Enfortumab vedotin | EV-103:dry eye(28.8%), blurred vision(6.8%), corneal disorders(5.5%) | / | [54] |
|  | EV-201:dry eye(23%), increased lacrimation(14%), blurred vision(15%) | / | [55] |
|  | EV-301:dry eye(15.9%), blurred vision(4.1%), corneal disorders(0.7%) | EV-301:dry eye(0.7%) | [56] |
|  | EV-302:dry eye(35.6%), blurred vision(11.1%), corneal disorders(2.2%) | / | [57] |
| Tisotumab vedotin | InnovaTV 201:conjunctivitis(42%), dry eye(24%), ulcerative keratitis(7%), blepharitis(5%), keratitis(5%), conjunctival ulcer(4%), blurred vision (4%), vital dye staining cornea present(4%), conjunctival disorder(2%), conjunctival hyperemia(2%), conjunctival scar(2%), corneal irritation(2%), corneal thinning(2%), erythema of eyelid(2%), eye irritation(2%), eye nevus(2%), meibomianitis(2%), ophthalmological examination abnormal(2%), punctate keratitis(2%), trichiasis(2%), visual acuity reduced(2%) | InnovaTV 201:conjunctivitis(2%) | [64], [65] |
|  | InnovaTV 204:conjunctivitis(26%), dry eye(23%), keratitis(11%), ulcerative keratitis(2%) | InnovaTV 204:ulcerative keratitis(2%) | [66] |
|  | Innovatv 206:conjunctivitis(17.6%), conjunctivitis allergic(5.9%), scleritis(5.9%), hordeolum(5.9%), blurred vision(5.9%) | / | [67] |
|  | InnovaTV301:conjunctivitis(31.2%), keratitis(15.6%), dry eye(13.2%), blepharitis(4.4%), eye discharge(4.4%), punctate keratitis(3.2%), blurred vision(3.2%), eye pruritis(2.8%), eye pain(2.4%), increased lacrimation(2.4%), cataract(2%) | InnovaTV301:keratitis(2%), punctate keratitis(0.4%) | [68] |
| Telisotuzumab vedotin | LUMINOSITY:blurred vision(54%), keratitis(11%), dry eye(5%) | LUMINOSITY:blurred vision(1.2%), keratitis(0.6%) | [75], [76] |
| Disitamab vedotin | Increased lacrimation(0.1-1%), blurred vision(0.1-1%), periorbital swelling(0.1-1%) | / | [77] |
| Belantamab mafodotin | DREAMM-1(Part1):blurred vision(29%), dry eye(24%), photophobia(11%), visual impaiment(11%), corneal deposits(3%), corneal disorder(3%), corneal irritation(3%), corneal oedema(3%), corneal opacity(3%), diplopia(3%), eye pain(3%), eye pruritus(3%), foreign body sensatlon in eyes(3%), keratitis(3%), keratopathy(3%), limbal stem cell deficiency(3%), photopsia(3%), punctate keratitis(3%)  DREAMM-1(Part2):blurred vision(46%), dry eye(34%), photophobia(23%), increased lacrimation (11%), keratitis(9%), eye pain(6%), keratopathy(6%), eye pruritus(3%), night blindness(3%) | DREAMM-1(Part1):blurred vision(3%), dry eye(3%)  DREAMM-1(Part2):dry eye(3%), keratitis(6%), eye pain(3%) | [78] |
|  | DREAMM-2:keratopathy or corneal epithelium changes(73%), blurred vision(26%)[including blurred vision, diplopia, reduced visual acuity, and visual impairment], dry eye(19%), exophthalmos(0.5%), glaucoma(0.5%), retinal vein occlusion(0.5%) | DREAMM-2:keratopathy or corneal epithelium changes(24%), blurred vision(3%)[including blurred vision, diplopia, reduced visual acuity, and visual impairment], dry eye(0.5%), exophthalmos(0.5%), glaucoma(0.5%), retinal vein occlusion(0.5%) | [79] |
|  | DREAMM-3:blurred vision(40%), visual acuity reduced(19%), keratopathy(12%), visual impairment(6%) | DREAMM-3:blurred vision(5%), visual acuity reduced(9%), keratopathy(4%), visual impairment(5%) | [80] |
| Loncastuximab tesirine | LOTIS-2:cataract(1%) | LOTIS-2:cataract(1%) | [91] |
| Gemtuzumab ozogamicin | Eye haemorrhage(1%), retinal haemorrhage(1%), conjunctival haemorrhage(1%), scleral haemorrhage(1%), eyelid hematoma(1%), eye swelling(0.4%), periorbital edema(0.4%) | Retinal haemorrhage(0.4%) | [92] |
| Inotuzumab ozogamicin | INO-VATE ALL:conjunctival hemorrhage, eyelid bleeding | / | [94], [95] |
| Trastuzumab deruxtecan | DESTINY-Breast01:dry eye(11%), periorbital edema, eyelid edema | DESTINY-Breast01:dry eye(0.4%) | [96] |
|  | DESTINY-Breast02:dry eye(6%), blurred vision(3%)[including blurred vision and visual impairment] | / | [97], [98] |
|  | DESTINY-Breast03:blurred vision(3.5%) | / | [98], [99] |
|  | DESTINY-Breast04:blurred vision(4.9%)[including blurred vision and visual impairment], eye hemorrhage | / | [98], [100] |
|  | DESTINY-Breast06:dry eye(7%), blurred vision(5%) | / | [98], [101] |
| Sacituzumab govitecan | IMMU-132-01:periorbital edema | / | [107], [108] |
| Datopotamab deruxtecan | TROPION-PanTumor01:  NSCLC cohort:ocular surface toxicity(32.2%)[dry eye, increased lacrimation, blepharitis];  Breast cancer cohort:ocular surface toxicity(HR+/HER2-:41.5%,TNBC:36.4%)[dry eye(HR+/HER2-:24.4%,TNBC:15.9%), keratitis(HR+/HER2-:9.8%,TNBC:2.3%)], cataract(1.2%) | TROPION-PanTumor01:  NSCLC cohort:keratitis(1.3%), ulcerative keratitis(1.5%) | [113], [114] |
|  | TROPION-Breast01:ocular surface toxicity(40%)[blepharitis, conjunctivitis, corneal disorder, corneal erosion, corneal lesion, dry eye, foreign body sensation in eyes, keratitis, keratopathy, increased lacrimation, limbal stem cell deficiency, meibomian gland dysfunction, ocular toxicity, photophobia, punctate keratitis, superior limbic keratoconjunctivitis, ulcerative keratitis, blurred vision, visual impairment, xerophthalmia], dry eye(21.7%), keratitis(14.4%), blepharitis(4.7%), increased lacrimation(6.4%), meibomian gland dysfunction(5.8%), conjunctivitis(2.8%), blurred vision(3.1%) | TROPION-Breast01:dry eye(0.6%), keratitis(0.6%) | [115] |
| Sacituzumab tirumotecan | KL264-01:dry eye(2.8%) | / | [116] |
|  | OptiTROP-Lung03:blurred vision(1.1%), ophthalmodynia(1.1%) | / | [117] |
|  | OptiTROP-Breast01:xerophthalmia(2.3%), blurred vision(1.5%) | / | [118] |
| Trastuzumab rezetecan | NCT04446260:ulcerative keratitis(2.2%) | NCT04446260:ulcerative keratitis(2.2%) | [119] |
|  | Blurred vision(1-10%), conjunctival hyperemia(0.1-1%), increased lacrimation(0.1-1%) | / | [120] |
| Moxetumomab pasudotox | NCT01829711:blurred vision(9%), dry eye(8%), cataract(5%), ocular discomfort/pain(4%), ocular swelling/periorbital edema(4%), conjunctivitis(1.3%), conjunctival hemorrhage(1.3%), ocular discharge(1.3%) | / | [121], [122] |
| Cetuximab saratolacan | NCT02422979:eye disorders(6.7%) | NCT02422979:periorbital edema(3.3%) | [123] |

ADC, antibody-drug conjugate; AEs, adverse events; NSCLC, non-small cell lung cancer; HR, hormone receptor; TNBC, triple-negative breast cancer.
