# Supplementary material for: Ocular toxicity associated with antibody-drug conjugates in cancer therapy: a comprehensive review
Source: Front Immunol. 2026 Apr 27;17:1698458. doi: 10.3389/fimmu.2026.1698458 (PMC13158211; doi:10.3389/fimmu.2026.1698458)
Supplement: Supplementary file 3 [file Table3.docx]

**Supplementary Material 3**

Supplementary Table 3 Postmarketing ocular AEs associated with ADCs.

| ADC | Ocular AEs | Reference |
| --- | --- | --- |
| Ado-Trastuzumab emtansine | Excessive eye blinking(IC025=4.00), increased lacrimation(IC025=3.02), cataract(IC025=0.38), blindness(IC025=0.59), retinal detachment(IC025=2.68), asthenopia(IC025=2.29), corneal disorder(IC025=0.73), ulcerative keratitis(IC025=0.17), abnormal sensation in eye(IC025=1.15), scintillating scotoma(IC025=1.92), hypermetropia(IC025=0.49), corneal deposits(IC025=0.78) | [38] |
|  | Corneal disorder(IC025=0.9), hypermetropia(IC025=0.11), corneal deposits(IC025=0.2), eye haemorrhage(n=8) | [39] |
|  | Dry eye(IC025=1.41) | [40] |
|  | Increased lacrimation | [42] |
|  | Blurred vision, diplopia | [43] |
| Mirvetuximab soravtansine | Visual acuity reduced(grade 3:16%), blurred vision(grade 3:16%) | [48] |
|  | Blurred vision, dry eye, keratitis, cataract | [50] |
|  | Corneal toxicity | [51] |
| Polatuzumab vedotin | Age-related macular degeneration(IC025=2.36), choroidal neovascularisation(IC025=0.01) | [38] |
| Enfortumab vedotin | Increased lacrimation(IC025=0.69), blurred vision(IC025=0.36), dry eye(IC025=2.32), keratitis(IC025=0.34), eye discharge(IC025=1.33), corneal disorder(IC025=1.41), eye haemorrhage(IC025=0.53), abnormal sensation in eye(IC025=1.13), blepharitis(IC025=0.66), xeropthalmia(IC025=0.81), giant papillary conjunctivitis(IC025=0.69) | [38] |
|  | Keratitis(IC025=2.34) | [59] |
|  | Eye disorders(grade1-2, 5.6%)[dry eye, conjunctivitis, abducens paresis] | [60] |
|  | Blurred vision(15%) | [61] |
|  | Eye disorders(10.2%) | [62] |
|  | Eye disorders(grade1-2, 8.8%)[dry eye, conjunctivitis, abducens paresis] | [63] |
| Tisotumab vedotin | Blurred vision(IC025=1.22), cataract(IC025=1.28), dry eye(IC025=4.00), ocular hyperaemia(IC025=2.34), keratitis(IC025=3.68), eye disorder(IC025=2.89), eye discharge(IC025=2.18), corneal disorder(IC025=2.20), visual acuity reduced(IC025=0.19), ulcerative keratitis(IC025=3.39), punctate keratitis(IC025=3.46), eye pain(IC025=0.81), conjunctival hyperaemia(IC025=1.25), ectropion(IC025=1.39), symblepharon(IC025=1.89), corneal deposits(IC025=1.39), corneal opacity(IC025=0.68), conjunctival ulcer(IC025=0.73) | [38] |
|  | Conjunctival subepithelial fibrosis, pseudomembranous conjunctivitis, conjunctival scarring, infectious keratitis | [69] |
| Brentuximab vedotin | Blindness(IC025=0.62), blindness unilateral(IC025=0.41), papilloedema(IC025=1.42), retinal haemorrhage(IC025=0.84), visual acuity reduced transiently(IC025=1.68) | [38] |
|  | Uveitis | [70] |
|  | Panuveitis | [71] |
|  | Vogt-Koyanagi-Harada-like granulomatous pan uveitis | [72] |
|  | Purtscher-like retinopathy | [73] |
|  | Retinal detachment(ROR=7.28) | [74] |
| Belantamab mafodotin | Keratopathy(any grade:68.4%, grade≥3:40.5%), blurred vision(any grade:36.8%, grade≥3:6.3%), dry eyes(4.7%) | [81] |
|  | Keratopathy(any grade:71.4%, grade 3: 54%) | [82] |
|  | Keratopathy(any grade:69%, grade≥3:30% ), visual acuity reduced(32%) | [83] |
|  | Keratopathy(grade≥3:11.1%), visual acuity reduced(26%), conjunctival hemorrhage(3.7%), xerophthalmia(14.8%) | [84] |
|  | Keratopathy(81.3%), visual acuity reduced(4.8%) | [85] |
|  | Keratopathy/keratitis(any grade:41.8%, grade≥3:8.2%), visual acuity reduced(any grade:10.9%, grade≥3:1.1%), dry eye(grade≥3:2.2%) | [86] |
|  | Blurred vision(30.3%), dry eye(24.2%), foreign body(9.1%), vision loss(6.1%), photophobia(3%), keratopathy(any grade:51.5%, grade≥3:21.2%), visual acuity reduced(30.3%) | [87] |
|  | Keratopathy(41.3%), blurred vision(28.3%), dry eye(17.4%), keratitis(9.8%) | [88] |
|  | Visual acuity reduced, epithelial crystal-like deposits | [89] |
|  | Blurred vision, microcystic keratopathy | [90] |
| Gemtuzumab ozogamicin | Retinal haemorrhage(IC025=0.08) | [38] |
|  | Ocular bleeding(grade 4:11.1%,) | [93] |
| Inotuzumab ozogamicin | Conjunctival haemorrhage(IC025=1.72), eyelid oedema(IC025=1.04) | [38] |
| Trastuzumab deruxtecan | Excessive eye blinking(IC025=4.00), keratitis(IC025=1.43), corneal disorder(IC025=0.10), punctate keratitis(IC025=1.56), eye haematoma(IC025=0.77), pinguecula(IC025=1.25) | [38] |
|  | Dry eye(ROR=4.53), corneal disease(ROR=24.34), blindness(ROR=2.47), keratitis(ROR=21.95) | [105] |
|  | Eye hematoma(ROR=49.81) | [106] |
| Sacituzumab govitecan | Cataract(IC025=0.05), eyelid ptosis(IC025=2.09) | [38] |
|  | Periorbital oedema(IC025=0.53) | [110] |
|  | Periorbital oedema(IC025=7.98) | [111] |
|  | Cataracts, dry eyes, ulcerative keratitis, increased lacrimation, visual impairment, papilledema of optic nerve, photophobia, eye congestion, eyelid disease, periorbital edema | [112] |

ADC, antibody-drug conjugate; AEs, adverse events; IC025, the lower bound of the 95% confidence interval for information component; ROR, reporting odds ratio.
